# *Myrtoessa hyas*, a new valvatiform genus and a new species of the Hydrobiidae (Caenogastropoda, Truncatelloidea) from Greece

**DOI:** 10.3897/zookeys.640.10674

**Published:** 2016-12-13

**Authors:** Canella Radea, Aristeidis Parmakelis, Sinos Giokas

**Affiliations:** 1 Department of Ecology and Systematics, Faculty of Biology, National and Kapodistrian University of Athens, 15784 Panepistimiopolis, Greece; 2 Department of Biology, University of Patras, 26500, Patras, Greece

**Keywords:** Endemicity, freshwater diversity, hydrobiids, taxonomy

## Abstract

A new to science valvatiform hydrobiid, *Myrtoessa hyas* Radea, **gen. n. & sp. n.**, from southern Greece, is described and illustrated. The new genus is a tiny gastropod thriving in a stream and is differentiated from the other known European and circum-Mediterranean valvatiform hydrobiid genera by a unique combination of the male and female genitalia features i.e. penis long, flat, blunt, with wide wrinkled proximal part and narrow distal part with a sub-terminal eversible papilla on its left side, bursa copulatrix well-developed, pyriform, fully protruding from the posterior end of the albumen gland and two seminal receptacles respectively. The new monotypic and locally endemic genus is narrowly distributed and its single known population nearby a coastal bustling village is vulnerable to anthropogenic stressors.

## Introduction

The freshwater fauna around the Mediterranean Basin comprises a plethora of valvatiform hydrobiids (Bodon et al. 2001). Many of them still have unclear taxonomic status because they were established on the basis of shell characters, which are often convergent, and/or those anatomical characters which are frequently non-diagnostic as for instance, stomach (Arconada and Ramos 2006). However, a more detailed anatomical description of some already known valvatiform taxa initially established from shell characters elucidated their taxonomic status (e.g. Bodon et al. 2001, Arconada and Ramos 2002, 2006).

Moreover, during last ten years, several new valvatiform taxa have been described based on shell and diagnostic anatomical characters and, in several cases, their molecular affinities have been investigated (e.g. Bodon et al. 2001, Arconada and Ramos 2006, Arconada and Ramos 2007, Arconada et al. 2007, Radea 2011, Rolan and Pardo 2011, Falniowski and Szarowska 2011a,b, Callot-Girardi and Boeters 2012, Radea et al. 2013).

In Greece, eight valvatiform-planispiral hydrobiid genera, namely *Daphniola* Radoman, 1973, *Fissuria* Boeters, 1981, *Graecoarganiella* Falniowski & Szarowska, 2011a, *Hauffenia* Pollonera, 1898, *Isimerope* Radea & Parmakelis, 2013, *Islamia* Radoman, 1973, *Prespolitorea* Radoman, 1983 and *Pseudoislamia* Radoman, 1979, have been recorded so far (Schütt 1980, Radoman 1983, Reischütz and Reischütz 2004, Falniowski and Szarowska 2011a, Radea et al. 2013). Three of them, i.e. *Pseudoislamia*, *Graecoarganiella* and *Isimerope*, are Greek endemics with a rather limited distribution in a few localities of Etoloakarnania, Phokida (central Greece), Argolida and Arkadia (Peloponnisos, southern Greek mainland).

Herein, a new genus and a new species of a minute valvatiform hydrobiid gastropod collected from Mount Parnon, Arkadia are described, and an identification key provided for the valvatiform hydrobiid genera of Greece based on the character states of male and female genitalia.

## Materials and methods

**Figure 1.**
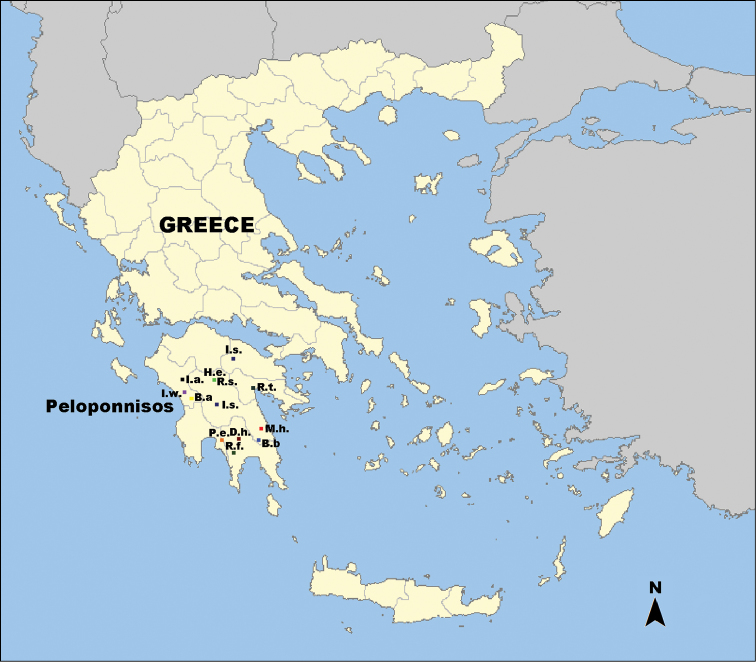
Map showing the distribution of the locally endemic Truncatelloidea in Peloponnisos, southern Greek mainland. Abbreviations: B.a. *Bythinella atypicos*, B.b. *Bythinella beckmanni*, D.h. *Daphniola hadei*, H.e. *Hauffenia edlingeri*, I.a. *Iglica alpeus*, I.s. *Isimerope semele*, I.w. *Iglica wolfischeri*, M.h. *Myrtoessa hyas* gen. n., sp. n., P.e. *Pseudamnicola exilis*, R.f. *Radomaniola feheri*, R.s. *Radomaniola seminula*, R.t. *Radomaniola tritonum*.

Snails in question thrived in a stream at Poulithra village, Parnon Mt., Arkadia (Fig. 1); GPS coordinates were taken using a hand-held unit (Magellan Triton 2000). Specimens were collected by hand from stones, gravel, mosses, and dead leaves. Immediately after collection, the specimens were placed into vials filled with water from the collection site and were transported alive to the lab. A digital picture using a camera (Canon EOS 1000D) attached on a stereomicroscope (Stemi 2000-C, Zeiss, Germany), was taken from each sample prior to the addition of any tissue preservation substances.

General and diagnostic shell characters were studied and four shell measurements (shell height and width, aperture height and width) were taken from 14 specimens using the micrometer of the Stemi 2000-C stereomicroscope. Four ratios were generated from the raw data (Sh/Sw, Ah/Aw, Sh/Ah and Sw/Aw).

Ten specimens were dissected and studied anatomically under the stereomicroscope using very fine pins and pointed watchmaker’s forceps. Prior to dissection, the shell of each specimen was removed by soaking in Pereny solution. The soft body features were documented using the digital camera as described above.

To remove tissue remaining and debris, the shell, the radula and the operculum were immersed in KOH solution (5g/l) at room temperature, rinsed in distilled water and air-dried before being mounted on stubs. The protoconch, the operculum and the radula were studied using scanning electron microscopy (SEM, Jeol JSM–35 operating at 25 kV) after being dried and spray-coated in gold–palladium.

The authority of the family Hydrobiidae was based on Bouchet and Rocroi (2005). In the description of the morphological characters and their states, the terminology of Hershler and Ponder (1998) was adopted.

A restricted number of specimens (27 specimens in total) was collected from the sampling locality because the population abundance seemed to be low (no specimen was found during the initial 5 min sampling effort). The collected material was deposited in the Zoological Museum (**ZMUA**) of the National & Kapodistrian University of Athens (**UOA**) and in the personal collection of C. Radea deposited in the Department of Ecology & Systematics, UOA.

### Abbreviations

#### Shell characters:

Ah
 aperture height 


Aw
 aperture width 


CV* (1+1/4n)*SD/x
 coefficient of variation corrected for sample size (Sokal and Rohlf 1995) 


Max
 maximum 


Min
 minimum 


n
 number of specimens 


SD
 standard deviation 


Sh
 shell height 


Sw
 shell width 


x
 mean 


#### Anatomical characters:

Bc
 bursa copulatrix 


Bd
 bursal duct 


Cg
 capsule gland 


Cm
 commissure 


E
 eye 


Ec
 egg capsule 


Fp
 faecal pellets 


In
 intestine 


Lpg
 left pleural ganglion 


Md
 mantle 


O
 renal oviduct 


Oe
 oesophagous 


Ol
 oviduct loop 


P
 penis 


Pd
 penial duct 


R
 rectum 


Rcg
 right cerebral ganglion 


Sbg
 suboesophageal ganglion 


Sh
 shell 


Sn
 snout 


Sp
 Sub-terminal penial papilla 


Sr1
 distal seminal receptacle 


Sr2
 proximal seminal receptacle 


Ss
 style sac 


St
 stomach 


T
 tentacle 


V
 ventral channel 


## Systematic description

### Family Hydrobiidae Stimpson, 1865

#### Myrtoessa

Taxon classificationAnimaliaLittorinimorphaHydrobiidae

Radea gen. n.

http://zoobank.org/B85FE216-9EB4-46A2-AC80-206D8C5DC296

##### Type species.

*Myrtoessa hyas* sp. n. by original designation.

##### Diagnosis.

Shell minute (maximum height 1.05 mm, maximum width 1.30 mm), valvatiform with more or less depressed spire; operculum without peg; central tooth with one basal cusp on each side; ctenidium and osphradium present; penis long, flat, blunt, with wide wrinkled proximal part and narrow distal part with a sub-terminal eversible papilla; female genitalia with large pyriform bursa copulatrix, renal oviduct non-pigmented, coiled in an ε (Greek)- shape; two seminal receptacles lying parallel on the renal oviduct and rather close to each other, a small distal receptacle (Sr1) and a larger proximal one (Sr2).

##### Etymology.

The generic name derives from the Greek mythology: Myrtoessa (*Μυρτώεσσα* in Greek) was a naiad nymph in Arkadia. Gender feminine.

#### Myrtoessa hyas

Taxon classificationAnimaliaLittorinimorphaHydrobiidae

Radea sp. n.

http://zoobank.org/4811DC7A-037A-4D3A-9F02-36F5F3B2A2BF

**Figure 2.**
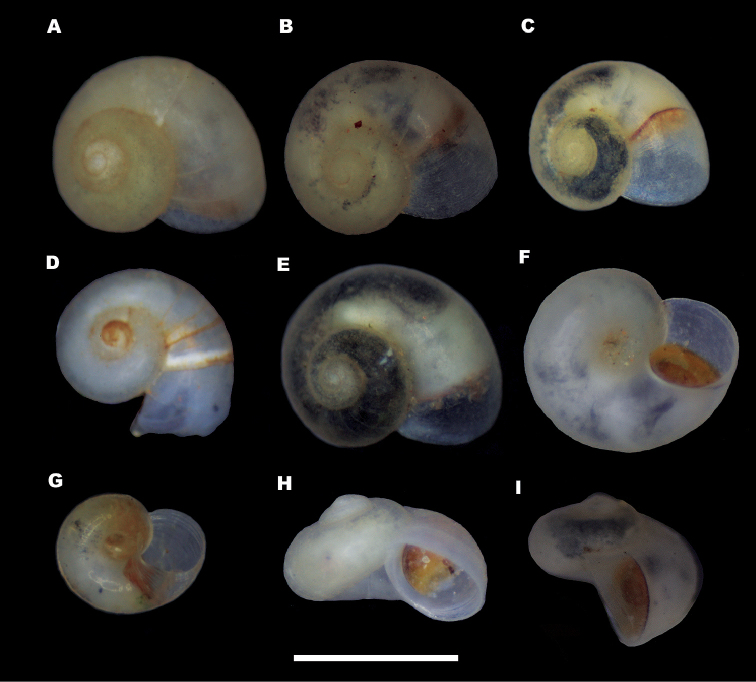
Shells of *Myrtoessa hyas* gen. n., sp. n. **A–E** Dorsal view **F–G** Ventral view **H-I** Lateral view. Scale bar 1 mm.

**Figure 3.**
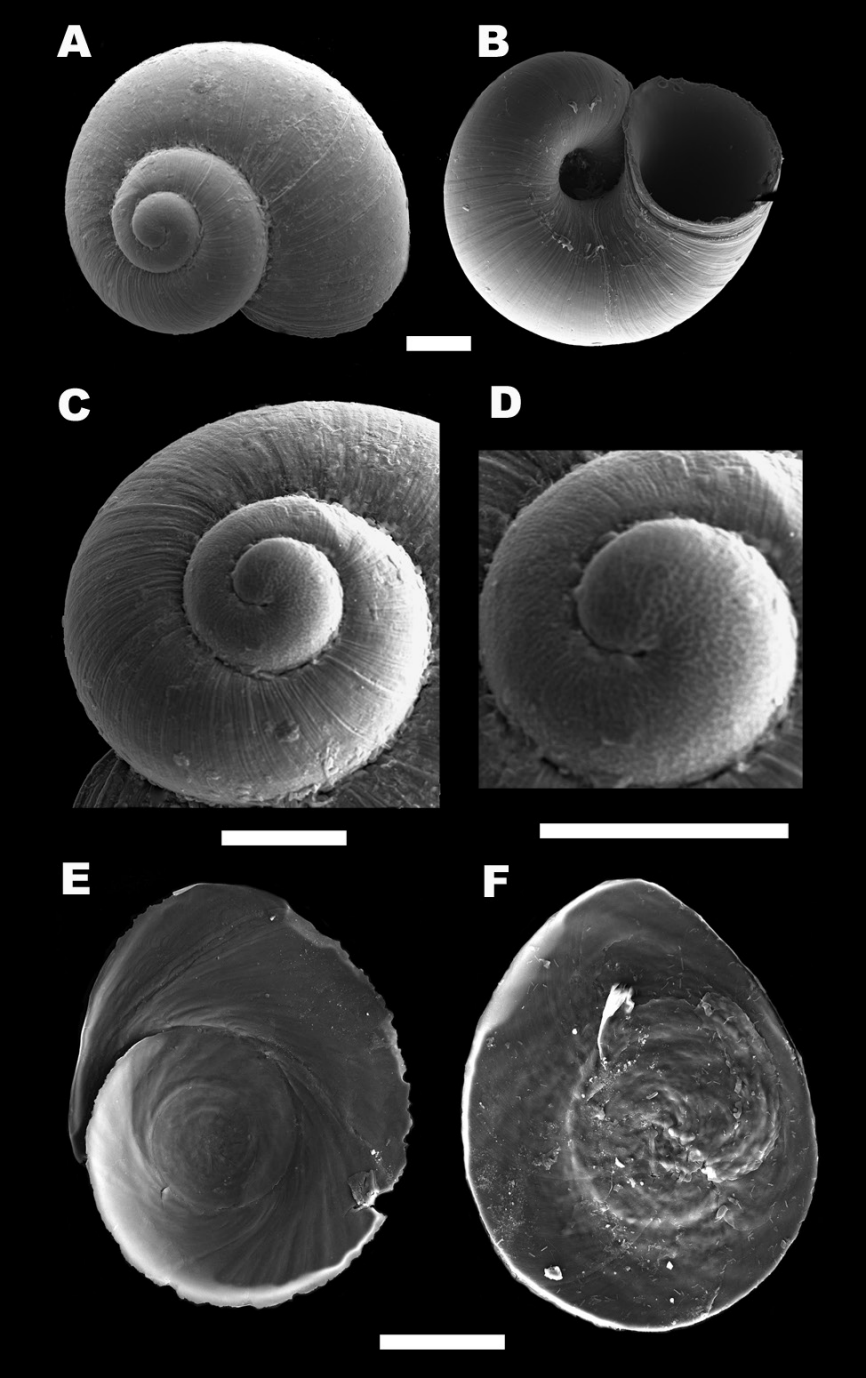
Scanning electron micrographs of shell, protoconch and operculum of *Myrtoessa hyas* gen. n., sp. n. **A–B** Shell dorsal and ventral view respectively **C** Shell apex showing protoconch **D** Close up of protoconch **E** Operculum, outer side **F** Operculum, inner side. Scale bars **A–D** 200 µm, **E–F** 100 µm.

**Figure 4.**
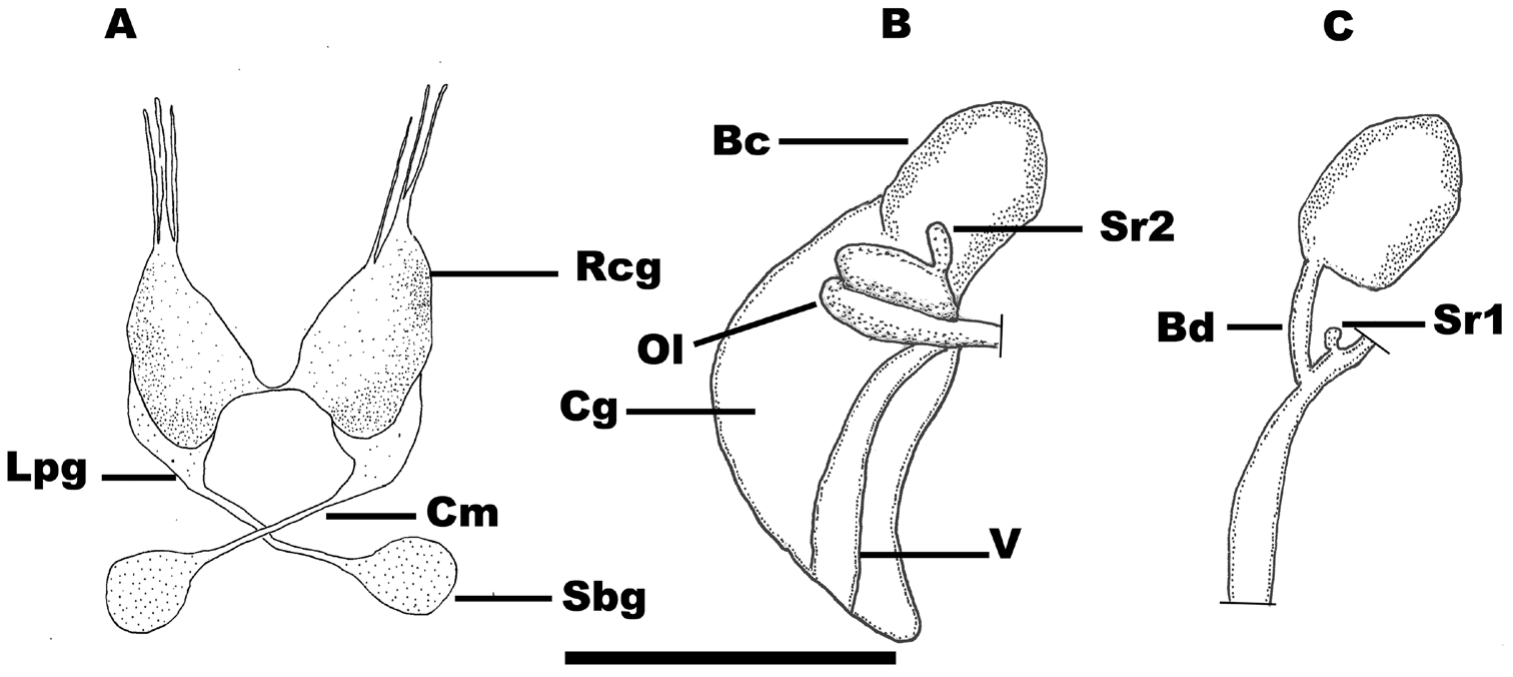
Nervous system and female genitalia of *Myrtoessa hyas* gen. n., sp. n. **A** Nervous system **B–C** Female genitalia. Scale bar 0.25 mm. Abbreviations are given in the Material and Method section.

**Figure 5.**
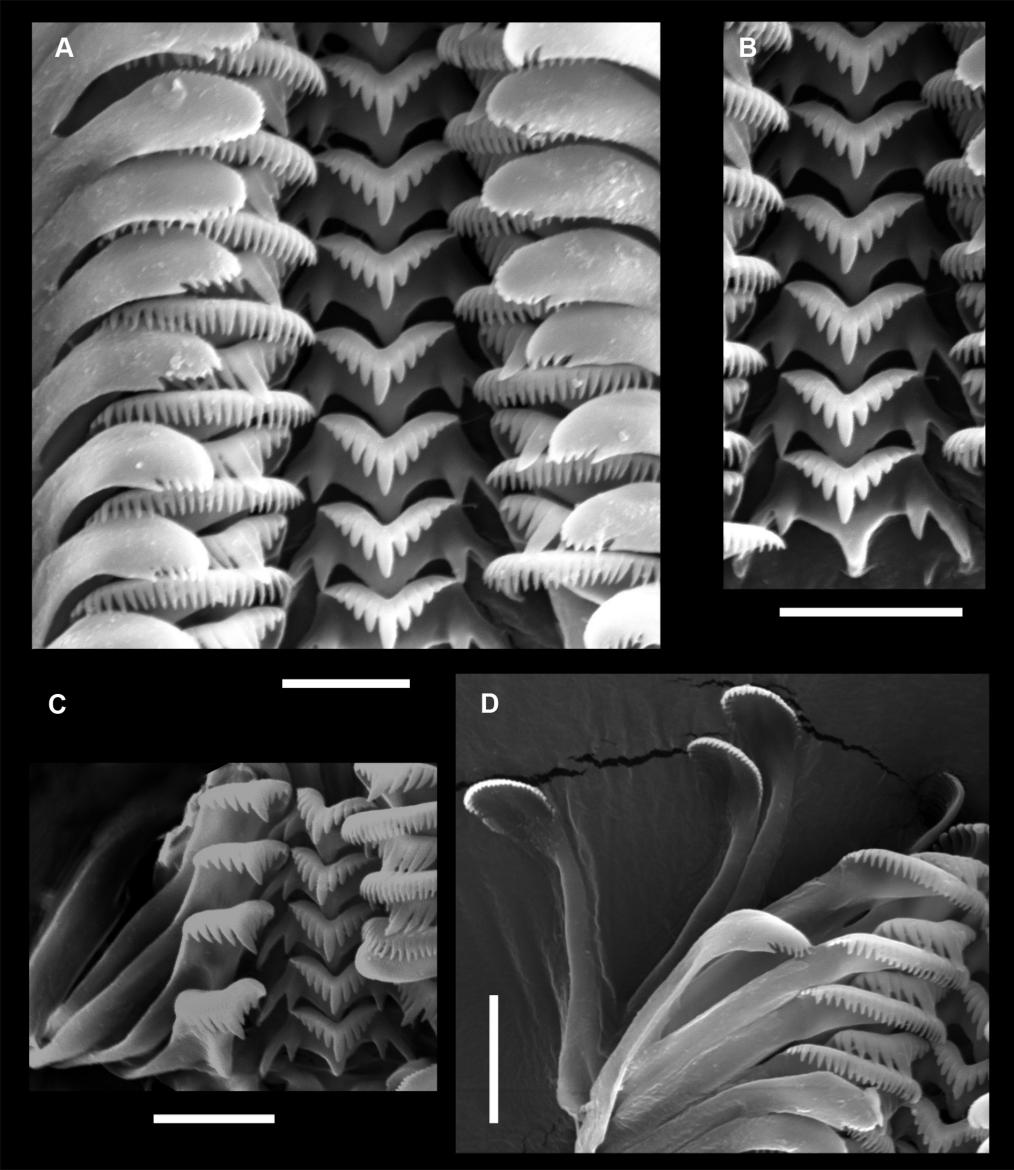
Scanning electron micrographs of radula of *Myrtoessa hyas* gen. n., sp. n. **A** Portion of radular ribbon **B** Central teeth **C** Lateral teeth **D** Inner and outer marginal teeth. Scale bars 10 µm.

**Figure 6.**
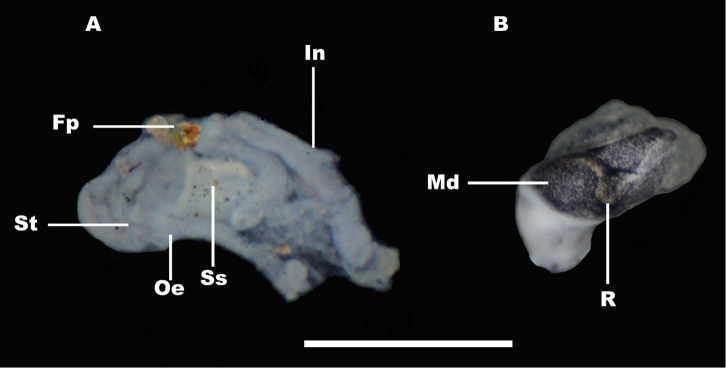
Digestive system (apart from the radula) of *Myrtoessa hyas* gen. n., sp. n. **A** Stomach, style sac, part of intestine and oesophagous **B** Rectum. Scale bar 0.5 mm. Abbreviations are given in the Material and method section.

**Figure 7.**
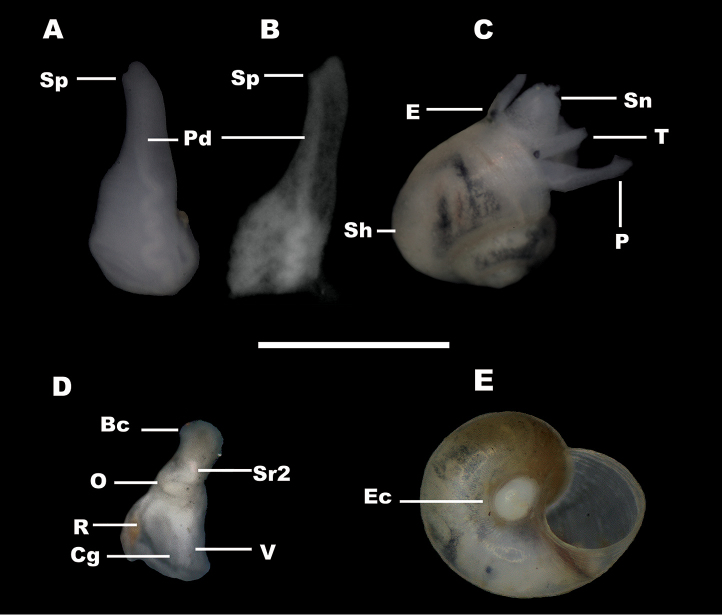
Reproductive anatomy of *Myrtoessa hyas* gen. n., sp. n. **A–C** Penis **D** Female genitalia (viewed from the left side) **E** Shell with an egg capsule into the umbilicus. Scale bars: **A, B, D** 0.5 mm, **C, E** 1 mm. Abbreviations are given in the Materials and methods.

Figs 2 , 3 , 4 , 5 , 6 , 7

##### Type-locality.

Poulithra, Peloponnese, Greece, 36°6.63'N, 22°53.53'E, 70 m a.s.l, stream, 12/IV/2014, C. Radea, G. Tryfonopoulos legs.

##### Diagnosis.

As for genus.

##### Etymology.

The specific name (in apposition) derives from the Greek mythology: Hyas, (Υάς in Greek), was one of the seven nymphs Hyades (Υάδες in Greek) bringing humidity and rain, daughters of Atlas and Pleione.

##### Type material.

**Holotype.** Ethanol-fixed specimen, ZMUA 4183.

**Paratypes.** Two ethanol-fixed specimens, ZMUA 4184. Ten ethanol-fixed specimens dissected for anatomical study and four specimens coated for SEM, the remaining in the personal collection of C. Radea deposited in the Department of Ecology & Systematics, UOA.

##### Other material examined.

Ten specimens, collected from the type locality, Th. Constantinidis, E. Kalpoutzakis legs, 25/IV/2014, in the personal collection of C. Radea deposited in the Department of Ecology & Systematics, UOA.

##### Description.

*Shell* (Fig. 2A–I). Colourless valvatiform shell with up to 3.5 whorls, thin, transparent when fresh, therefore possible to follow the position of rectum; spire more or less depressed; whorls rounded, regularly growing with shallow sutures. Measurements are given in Table 1. Periostracum cream-coloured; aperture adhering to the last whorl, prosocline, roundish to ovate; peristome continuous, thickened at columellar margin, reflected at columellar margin, the outer margin simple; umbilicus open, deep, wide so that the first whorls can be seen through it, sometimes partially covered by the collumelar margin of aperture (Figs 2F, G, 3B); protoconch microsculpture composed of a dense net of irregularly shaped depressions (Fig. 3A, C, D). The number of protoconch whorls is 1.25. The width of nucleus and protoconch is 102 µm and 262 µm, respectively.

**Table 1.** Shell morphometry of *Myrtoessa hyas* gen. n., sp. n. Measurements are in mm. Abbreviations are given in the materials and methods.

Type locality		Sh	Sw	Ah	Aw	Sh/Sw	Ah/Aw	Sh/Ah	Sw/Aw
Poulithra	Min	0.60	1.20	0.60	0.60	0.46	0.92	1.00	0.92
*N* = 14	Max	1.05	1.40	0.70	0.70	0.77	1.17	1.67	1.17
	*x*	0.88	1.31	0.63	0.63	0.67	1.02	1.40	1.02
	SD	0.12	0.09	0.04	0.05	0.08	0.08	0.19	0.08
	CV*	0.14	0.07	0.06	0.09	0.12	0.08	0.14	0.08

*Operculum* (Fig. 3E, F). Operculum ovate, thin, corneous, paucispiral, yellowish-orange, darker at the nucleus, with weakly convex inner face without any peg, nucleus sub-central.

*Soft body pigmentation* (Fig. 2A–I). Soft body pigmentation of alive specimens extremely variable, the colouration being visible under the transparent shell; many specimens almost totally unpigmented with only a few traces of pigments on walls of visceral sac, several specimens grey pigmented and some others dark grey pigmented; in the last two cases, tentacles with a median grey stripe and snout with grey areas laterally and around eyes; snout longer than wide, parallel-sided with medium distal lobation; eye spots present; tentacles about six times as long as wide (in specimens preserved in ethanol solution 70%).

*Nervous system* (Fig. 4A). Cerebral ganglia of the same size, white-coloured; supraoesophageal and suboesophageal ganglia of the same size, smaller than cerebral ganglia, white-coloured; supraoesophageal connective about equal to suboesophageal connective; mean RPG ratio 0.39 (three specimens), nervous moderately concentrated.

*Ctenidium*-*Osphradium*. Ctenidium with ca 5–7 long lamellae. Osphradium of intermediate width, opposite posterior part of ctenidium.

*Radula* (Fig. 5). Central tooth trapezoidal, dorsal edge of tooth strongly concave; one pair of medium-sized basal cusps (bc2), basal tongue broadly V-shaped and about equal to lateral margin; median cusp blunt, protruding, broader and longer than laterals, 5 lateral cusps on each side of median cusp, the latter one not well defined (Fig. 5A, B); lateral tooth face taller than wider, basal tongue well developed; outer wing moderately flexed; cutting edge much shorter than outer wing; central cusp longer than lateral cusps, 5 lateral cusps on outer side, 4-5 on inner side (Fig. 5C); inner marginal tooth with *ca.* 24-28 long almost equal in size cusps; outer marginal tooth with *ca.* 27 cusps (Fig. 5D).

*Digestive system* apart from radula (Fig. 6). Style sac smaller than stomach, not protruding to the intestinal loop (Fig. 6A); rectum V-shaped, V being wider in female specimens (Fig. 6B).

*Male reproductive system* (Fig. 7A–C). Penis long, tapering, flat, blunt, distal portion being well demarcated from proximal portion, opening through sub-terminal papilla on the left, whitish with a median grey stripe at the distal portion (in the grey pigmented specimens), proximal portion bent upon itself and wrinkled near the base; base usually black pigmented ventrally, its attachment area well behind the right eye; penial duct strongly undulating in base and straight distally, near centrally positioned and opening on the left side of penis; prostate like an elongate bean with mean length 0.44 mm (three specimens).

*Female reproductive system* (Figs 4B–C, 7D–E). Pallial oviduct glands, i.e. albumen and capsule glands, very small, total mean length 0.53 mm, total mean width 0.24 mm (three specimens); bursa copulatrix large-sized, pyriform, posteriorly positioned and fully protruding from the posterior end of the albumen gland; bursal duct length a little shorter than or equal to bursa copulatrix length; renal oviduct unpigmented and well-developed, tightly coiled in a shape of lower case ε (Greek); two seminal receptacles lying parallel on the renal oviduct and rather close to each other; distal seminal receptacle (Sr1) very small, globular with very short duct; proximal seminal receptacle (Sr2) larger, usually lying tightly over the renal oviduct and against bursa copulatrix; proximal seminal receptacle (Sr2) with a pink pearl shine. In some specimens, an egg capsule with a single egg was found inside the umbilicus (Fig. 7E).

##### Distribution and habitat.

So far the distribution of *Myrtoessa hyas* gen. n. & sp. n., seems to be restricted to the type locality on Parnon Mt., Peloponnisos. At the type locality, the geological substrate is limestone; all the specimens of the new species were found on stones, gravel, mosses and dead leaves of *Platanus orientalis* L. accumulated on the bottom of a stream. Many *Bythinella* sp. individuals were found to share the same stream.

## Discussion

Twelve locally endemic truncatelloidean species (see Reischütz and Reischütz 2004, 2008, Falniowski and Szarowska 2011b, Falniowski et al. 2012, Georgiev 2013, Radea et al. 2013a) have been described from Peloponnese so far (Fig. 1). The high number of endemic truncatelloideans was being expected since the complex topography and the intense geological history of this mainly mountainous area facilitate and promote the diversity and endemicity of invertebrates (Sfenthourakis and Legakis 2001, Legakis and Maragou 2009).

*Myrtoessa hyas* gen. n. & sp. n. differs from all the known valvatiform hydrobiids in having a unique combination of shell and anatomical characters that according to the standard hydrobiid taxonomy, does not allow its inclusion in any other known genus of the Hydrobiidae family. Consequently, a new monotypic genus is necessary to accommodate it.

The combination of the features of male and female genitalia followed by Bodon et al. (2001) for distinguishing the known genera of valvatiform Hydrobiidae shows that the new genus is clearly differentiated from the other European and circum-Mediterranean valvatiform genera (Table 2) having bursa copulatrix and two seminal receptacles by the penial characters. These genera are further differentiated from *Myrtoessa* gen. n. in having, among others, an operculum with peg (*Bracenica* Radoman, 1973, *Gocea* Hadzǐšiče 1956), no eyes (*Fissuria*, *Pezzolia* Bodon & Giusti, 1986, *Sardohoratia* Manganelli, Bodon, Cianfanelli, Talenti & Giusti, 1998), different shape of rectum (*Corbellaria* Callot-Girardi & Boeters, 2012, *Fissuria*, *Graecoarganiella*, *Horatia* Bourguignat, 1887, *Iberhoratia* Arconada & Ramos, 2007, *Pezzolia* Bodon & Giusti 1986, *Sardohoratia*).

**Table 2.** *Myrtoessa* gen. n. compared morphologically with other valvatiform genera distributed in the Balkan Peninsula and in the Mediterranean Basin: eleven morphological characters and character-state scores for thirty-four genera are given (based on Radea et al. 2013).

	Distribution	Bursa copulatrix	Seminal receptacle (s)	Penis	Penial lobe(s)	Penial papilla	Penial stylet	Ctenidium	Eyes	Operculum	Umbilicus	Rectum
*Arganiella*	Italy, Spain, Montenegro	1	1	0	0	0	0	1	0	0	2	(U) or (S)
*Boetersiella*	Spain	1	1	0	0	0	0	0	1	0	2	(U)
*Bracenica*	Montenegro	1	3	1	2	0	0	-	0	1	3	-
*Chondrobasis*	Spain	1	1	1	1	0	0	0	1	0	2	(U)
*Corbellaria*	Spain	1	3	1	2	0	0	0	-	0	2	(SS)
*Dabriana*	Bosnia	1	1	0	0	0	0	1	0	-	1	-
*Daphniola*	Greece	1	3	1	2	0	0	1	1	0	0	-
*Fissuria*	Greece, Italy, France	1	3	3	1+3	1	0	1	0	0	0,1,2,3	(S)
*Gocea*	FYROM	1	3	1*	4*	0	0	-	1	1	2	-
*Graecoarganiella*	Greece	1	3	1	1	1	0	0	1	-	2	(S)
*Hauffenia*	Italy, Greece	1	2	0,1	0,4	0	1	1, 0	0	1ª	2	(Z) or (?)
*Heraultiella*	France	1	1	0	0	0	0	1	0	0	2	(U) or (V)
*Horatia*	Croatia, FYROM	1	3	1,2	3	0	0	1	1	0	1	(0)
*Iberhoratia*	Spain	1	3	1	2	0	0	1	1	0	2	(U) or (S)
*Isimerope*	Greece	1	0	1	3	1	0	0	1	0	1	(U)
*Islamia*	Greece, France, Italy, Spain, Turkey, Israel	0	3	1	4	0	0	1	1	0	0	(U)
*Josefus*	Spain	0	3	1	4	0	0	0	1	0	1	(U)
*Karevia*	FYROM	1	3	1	3	0	0	-	1	0*	3	-
*Kerkia*	Slovenia	1	1	1	3	0	0	1	0	1	2	(S)
*Lyhnidia*	FYROM	1	2	1*	4*	0	0	-	1	0	0	-
*Milesiana*	Spain	0	3	1	2	0	0	1	1	0	2	(U)
*Myrtoessa*	Greece	1	3	0	0	2	0	1	1	0	2	(V)
*Ohridohauffenia*	FYROM	1	3	1	3	0	0	-	1	0	1	-
*Ohrigocea*	FYROM	1	3	1	3	0	0	-	1	0	2	-
*Pezzolia*	Italy	0,1	3	0	0	0	0	0	0	0	2	(S)
*Prespolitorea*	Greece, FYROM	1	3	1	3	0	0	-	1	0*	1	-
*Pseudohoratia*	FYROM	1	2	1	3	0	0	1	1	1	0,1,2	(0)
*Pseudoislamia*	Greece	1	3	1	4	0	0	-	1	0	2	-
*Sardohoratia*	Italy	1	3	0	0	0	0	0	0	0	0	(S)
*Sheitanok*	Turkey	1	1	0	0	0	0	1**	1	0*	3	-
*Spathogyna*	Spain	1	3	1	2	0	0	1	1	0*	2	(V)
*Strugia*	FYROM	1	2	1	3	0	0	-	1	0	2	-
*Tarraconia*	Spain	1	0	1	2	0	0	1	1	0	3	(U)
*Zaumia*	FYROM	1	2	1*	4*	0	0	-	0	0	1	-

Character states and symbols: **bursa copulatrix**: absent (0), present (1), **seminal receptacles**: absent (0), distal seminal receptacle (1), proximal seminal receptacle (2), distal and proximal seminal receptacle (3), **penis**: simple without lobe(s) (0), with one lobe (1), with two lobes (2), with more than two lobes (3), **penial lobe (s)**: absent (0), basal lobe (1), medial lobe (2), lobe at 2/3 of penis length (3), apical lobe (4), **penial papilla**: absent (0), present terminal eversible (1), present sub-terminal eversible (2) , **penial stylet**: absent (0), present (1), **ctenidium**: absent (0), present (1), **eyes**: absent (0), present (1), **operculum**: simple (0), peg-bearing (1), **umbilicus**: narrow (0), medium (1), wide (2), very wide (3), **rectum**: without or almost without bend (0), Z-like (Z), U-like (U), S-like (S), V-like (V), ?-like (?); ª: not present in all species *: it was deduced by Bodon et al. (2001), **: Schütt and Şessen 1989, page 117, fig 2B -: no data

Sources: Arconada and Ramos 2001, 2002, 2006, Arconada et al. 2007, Bodon and Giusti 1986, Bodon et al. 1995, Bodon et al. 2001, Boeters et al. 2014, Callot-Girardi and Boeters 2012, Falniowski and Szarowska 2011a, Girardi 2009, Giusti and Pezzoli 1981, Manganelli et al. 1998, Radea et al. 2013, Radoman 1966, 1983, Ramos et al. 2000, Schütt 1991, Schütt and Şessen 1989.

The opening of penial duct through a sub-terminal papilla is a novel character recorded for the first time in the valvatiform hydrobiids of Europe and Mediterranean Basin since, up to now, only a terminal papilla has been recorded (Table 2). The other valvatiform genera having a penial papilla, i.e. *Fissuria*, *Graecoarganiella* and *Isimerope*, are distinguished from *Myrtoessa* gen. n. by the position of the papilla, the different overall shape of the penis, the female genitalia (*Isimerope*) and other characters detailed in Table 2.

The new genus inhabits a stream with cold and clear fast running water. The rest known valvatiform genera of Greece thrive in various freshwater systems: *Islamia* and *Pseudoslamia* in lakes, springs and streams, *Isimerope* in springs and rivers, *Fissuria* in subterranean waters, *Daphniola*, *Hauffenia* and *Graecoarganiella* in springs and *Prespolittorea* in lakes (Radoman 1983, Reischütz 1988, 2004, Bodon et al. 2001, Falniowski and Szarowska 2011a, Radea et al. 2013a,b).

The single population of *Myrtoessa hyas* gen. n. & sp. n. nearby a coastal touristic village is vulnerable to anthropogenic stressors, in particular during the summer period, due to the numerous tourists, visitors, and hikers as well as to the increased demands for water supply and irrigation.

In last three years, one new locally endemic monotypic truncatelloidean genus, i.e. *Isimerope* (Radea et al. 2013), and seven new locally endemic species i.e. *Radomaniola feheri* Georgiev, 2013 (Georgiev 2013), *Daphniola magdalenae* Falniowski, 2015, *Iglica hellenica* Falniowski, 2015 (Falniowski and Sarbu 2015), *Pseudamnicola ianthe* Radea & Parmakelis, 2016, *Pseudamnicola ilione* Radea & Parmakelis, 2016 (Radea et al. 2016), *Pseudamnicola magdalenae* Falniowski, 2016 (Falniowski 2016a), and *Bythinella walensae* Falniowski, 2016 (Falniowski 2016b) were described from Greece. The introduction of one more new locally endemic genus provides clues about the richness and the high endemicity of Greek freshwater bodies, which support some of the most biodiverse and heavily threatened ecosystems of the Mediterranean Basin Biodiversity Hotspot (Szarowska and Falniowski 2004, Darwall et al. 2014).

### Key to the Greek valvatiform genera based on genitalia character states

**Table:** 

1	Bursa copulatrix present	**2**
–	Bursa copulatrix absent	***Islamia***
2	Seminal receptacle(s) present	**3**
–	Seminal receptacle(s) absent	***Isimerope***
3	Both proximal (Sr2) and distal (Sr1) receptacles present	**4**
–	Only proximal (Sr2) receptacle present	***Hauffenia***
4	Proximal seminal receptacle (Sr2) well developed, much larger than the distal one (Sr1)	***Prespolitorea***
–	Not as above	**5**
5	Penis with papilla	**6**
–	Penis without papilla	**8**
6	Penis with terminal papilla	**7**
–	Penis with sub-terminal papilla, without lobe(s), distal portion of penis well demarcated from proximal portion	***Myrtoessa***
7	Penis pigmented black, long, tapering, cylindrical with one double lobe on its proximal portion	***Graecoarganiella***
–	Penis unpigmented, rather short, parallel-sided, flat with more than one glandular lobes on distal, occasionally on proximal portion too	***Fissuria***
8	Penis with a wide lobe on its distal portion	***Pseudoislamia***
–	Penis with a narrow lobe on its proximal portion	***Daphniola***

## Supplementary Material

XML Treatment for Myrtoessa

XML Treatment for Myrtoessa hyas
